# Unraveling the intercellular communication disruption and key pathways in Alzheimer’s disease: an integrative study of single-nucleus transcriptomes and genetic association

**DOI:** 10.1186/s13195-023-01372-w

**Published:** 2024-01-02

**Authors:** Andi Liu, Brisa S. Fernandes, Citu Citu, Zhongming Zhao

**Affiliations:** 1 Department of Epidemiology, Human Genetics and Environmental Sciences, School of Public Health, The University of Texas Health Science Center at Houston, Houston, TX 77030 USA; 2 Center for Precision Health, School of Biomedical Informatics, The University of Texas Health Science Center at Houston, 7000 Fannin St., Suite 600, Houston, TX 77030 USA; 3 Faillace Department of Psychiatry and Behavioral Sciences, McGovern Medical School, The University of Texas Health Science Center at Houston, Houston, TX 77030 USA; 4 Department of Biomedical Informatics, Vanderbilt University Medical Center, Nashville, TN 37203 USA

**Keywords:** Alzheimer’s disease, Cell–cell communication, Multi-omics, Single-nucleus RNA sequencing

## Abstract

**Background:**

Recently, single-nucleus RNA-seq (snRNA-seq) analyses have revealed important cellular and functional features of Alzheimer's disease (AD), a prevalent neurodegenerative disease. However, our knowledge regarding intercellular communication mediated by dysregulated ligand-receptor (LR) interactions remains very limited in AD brains.

**Methods:**

We systematically assessed the intercellular communication networks by using a discovery snRNA-seq dataset comprising 69,499 nuclei from 48 human postmortem prefrontal cortex (PFC) samples. We replicated the findings using an independent snRNA-seq dataset of 56,440 nuclei from 18 PFC samples. By integrating genetic signals from AD genome-wide association studies (GWAS) summary statistics and whole genome sequencing (WGS) data, we prioritized AD-associated Gene Ontology (GO) terms containing dysregulated LR interactions. We further explored drug repurposing for the prioritized LR pairs using the Therapeutic Targets Database.

**Results:**

We identified 190 dysregulated LR interactions across six major cell types in AD PFC, of which 107 pairs were replicated. Among the replicated LR signals, we found globally downregulated communications in the astrocytes-to-neurons signaling axis, characterized, for instance, by the downregulation of *APOE*-related and Calmodulin (*CALM*)-related LR interactions and their potential regulatory connections to target genes. Pathway analyses revealed 44 GO terms significantly linked to AD, highlighting Biological Processes such as ‘amyloid precursor protein processing’ and ‘ion transmembrane transport,’ among others. We prioritized several drug repurposing candidates, such as cromoglicate, targeting the identified dysregulated LR pairs.

**Conclusions:**

Our integrative analysis identified key dysregulated LR interactions in a cell type-specific manner and the associated GO terms in AD, offering novel insights into potential therapeutic targets involved in disrupted cell–cell communication in AD.

**Supplementary Information:**

The online version contains supplementary material available at 10.1186/s13195-023-01372-w.

## Background

Alzheimer’s disease (AD) is a progressive neurodegenerative disease that affects over 32 million individuals worldwide, resulting in substantial societal and economic burden [1]. AD is characterized by extracellular deposits of β-amyloid and intraneuronal accumulation of neurofibrillary tangles, ultimately resulting in neuronal death [2]. Despite extensive research, with the exception of the amyloid deposition mechanism, the molecular and cellular mechanisms underlying AD remain elusive, which translates into limited effective therapies.

A delicate balance of intercellular communications among non-neuronal and neuronal cells is essential for maintaining tissue homeostasis and normal brain functions such as synaptic pruning and synaptogenesis [3–5]. Experimental and genetic evidence implicates the aberrant activation of microglia and astrocytes as contributing factors in the pathogenesis of neurodegenerative diseases. These activated cells exert downstream effects on neurons, further implicating them in diseases including AD [6–8]. Recent studies have leveraged single-nucleus RNA sequencing (snRNA-seq) data [9–11] and intercellular communication analysis tools [12, 13] to identify complex intercellular communication within the postmortem AD brains [14–16]. Most findings suggested that microglia might contribute to AD’s pathogenesis through ligand-receptor (LR) axis communication alterations [14–16]. For instance, it has been proposed that the amyloid-β peptides that bind to the receptor for advanced glycation end products could lead to upregulation of the *chromogranin A* gene, triggering neurotoxin production and inflammation in AD [8]. Another study has pointed to the activation of SEMA6D-TREM2 interaction in proximity to Aβ plaques, inducing microglial activation in AD [15]. Nevertheless, the disrupted interactions between astrocytes and neurons remain largely unexplored. Hence, a more thorough intercellular communication analysis using snRNA-seq data is crucial for deeper insights into the interplay between non-neuronal and neuronal cells in AD.

Understanding the biological relevance of dysregulated intercellular signals in AD requires a comprehensive assessment of their associated pathways [4]. An integrative analysis framework incorporating genetic variant data could enhance our understanding of biological pathways involving dysregulated communication signals in AD. Researchers have gained insight into atherosclerosis-associated biological mechanisms through snRNA-seq data-guided pathway-level polygenic scores (PGSs) analysis by integrating genome-wide association studies (GWASs) statistics and genotyping data [17]. However, this integrative approach has not yet been applied to AD snRNA-seq data. While previous snRNA-seq studies have mapped AD GWAS risk loci [18–21] to AD-associated genes and open chromatin regions of microglia, astrocytes, and oligodendrocytes [11, 22, 23], a more systematic integrative analysis is necessary to unveil the complex connections between genetic variants and specific pathways encompassing dysregulated communication signals in AD.

To address this gap, we engineered a comprehensive integrative analysis framework to reconstruct the dysregulated intercellular communication network and identify their underlying biological functions in AD. Specifically, we collected two human prefrontal cortex (PFC) snRNA-seq datasets from AD individuals and healthy controls of two independent cohorts, used as discovery and replication datasets [9, 11]. Through a systematic comparative intercellular communication analysis, we identified dysregulated LR pairs and their potential target genes across six major cell types, namely astrocytes, excitatory neurons, inhibitory neurons, microglia, oligodendrocytes, and oligodendrocyte precursor cells (OPCs). Secondly, we conducted pathway-level analyses, leveraging GWAS statistics and genotyping data of AD participants and healthy controls, to prioritize biological pathways containing dysregulated communication signals. Lastly, our drug repurposing analysis, utilizing publicly available databases, revealed known and novel repurposable drugs for AD treatment. This study provides novel insights into the complex intercellular communication in AD postmortem brains, suggesting potential molecular mechanisms and therapeutic strategies for AD.

## Methods

### snRNA-seq data for AD

We collected two snRNA-seq datasets for AD research, one for discovery and the other for replication. The discovery dataset comprised postmortem human brain samples from 48 participants, sourced from The Religious Orders Study and Memory and Aging Project (ROSMAP) cohort. This dataset included 24 AD samples and 24 sex/age of death-matched control samples. The classification of AD and controls was determined based on pathological hallmarks of AD, including β-amyloid burden, neuritic plaque burden, and global AD-pathology burden [9]. Droplet-based snRNA-seq data were generated from the PFC region of these 48 samples, resulting in transcriptome profiles for 80,660 single nuclei [9]. The count matrix, mapped by using Cell Ranger (v.2.0.0, GRCh38.p5 reference genome), was downloaded from the AD Knowledge Portal [24].

The independent replication dataset consists of postmortem human brain samples from 11 AD participants and 7 healthy controls, all from the University of California Irvine Institute for Memory Impairments and Neurological Disorders (UCI MIND) Alzheimer’s Disease Research Center (ADRC) [11]. The diagnosis of AD was defined based on the Braak and plaque staging [11]. The snRNA-seq data were generated from 61,472 isolated nuclei from the PFC region of these 18 individuals. We retrieved the data from the National Center for Biotechnology Information Gene Expression Omnibus (GSE174367).

### snRNA-seq data quality control and annotation

**Fig. 1:**
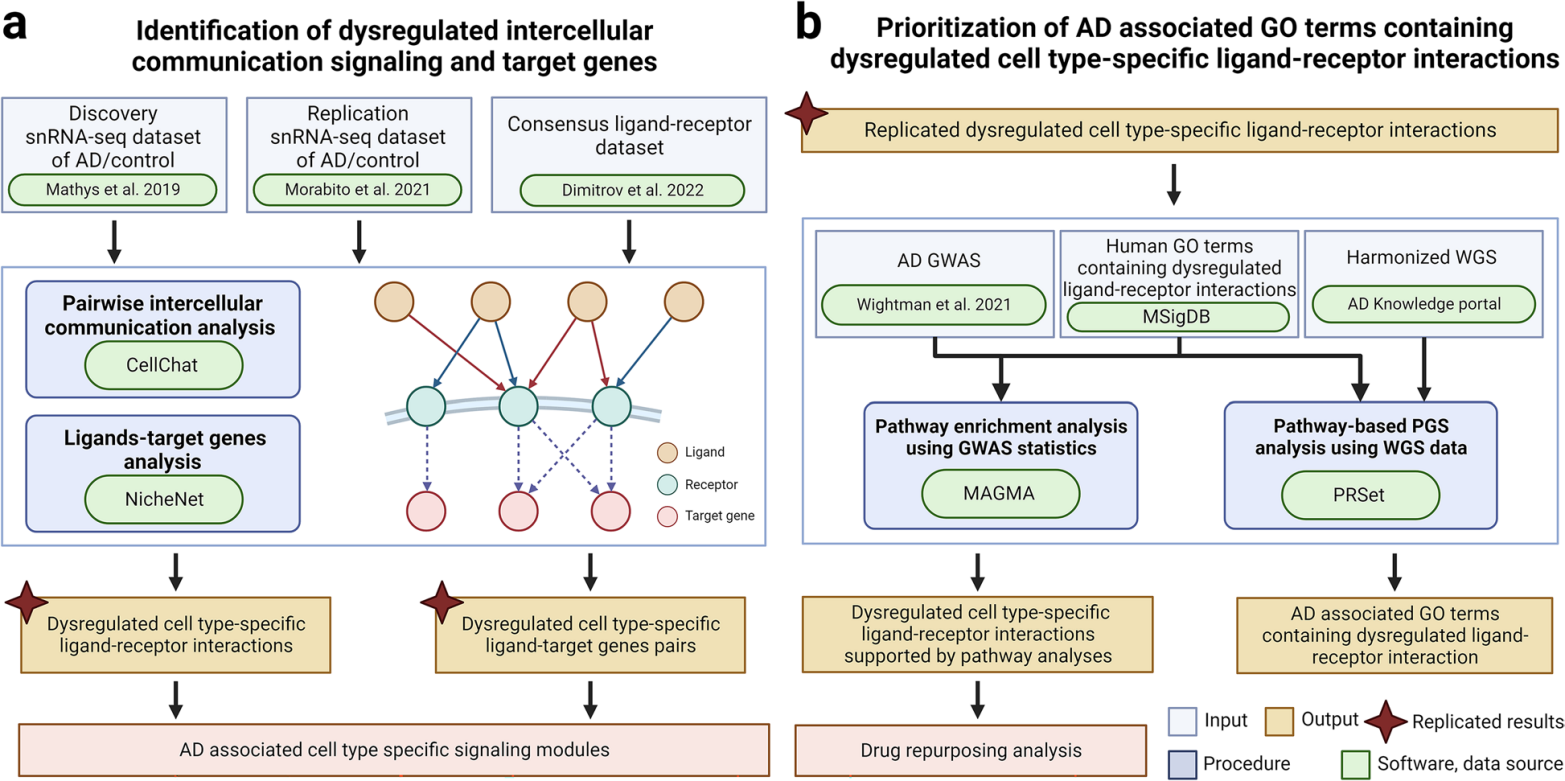
Study workflow. **a** Identification of altered cell type-specific ligand-receptor (LR) pairs through systematic comparative cell–cell communication analysis in single nuclei RNA sequencing data of Alzheimer’s disease (AD) individuals and controls in the discovery and replication datasets. **b** Prioritization of altered cell type-specific LR pairs through pathway analyses by integrating genome-wide association studies (GWAS) summary statistics and whole genome sequencing (WGS) data

The integrative analysis framework is depicted in Fig. 1. We implemented universal preprocessing and quality control procedures for both the discovery and replication snRNA-seq datasets, starting from the count matrix, using the standard Seurat pipelines (v.4.3.0) [25]. Specifically, we retained cells containing between 200 and 6000 features, with mitochondrial reads constituting less than 5% of the total reads. We then applied the standard log-normalization workflow to the gene expression matrix via the *NormalizeData* function in Seurat. Dimensionality reduction was executed using the Uniform Manifold Approximation and Projection technique, and visual representation was confined to the initial two dimensions. We assigned cellular labels to eight major cell types in the brain, namely astrocytes, endothelial cells, excitatory neurons, inhibitory neurons, microglia, oligodendrocytes, OPCs, and pericytes, based on statistical enrichment of marker gene sets as delineated in the original publication [11, 25]. Endothelial cells and pericytes were excluded from the analysis due to their low cell count in both datasets.

### Intercellular communication analysis

To assess dysregulated intercellular communication signals among various cell types in the snRNA-seq dataset, we employed CellChat (version 1.6.0, last update on November 12, 2022). This approach allowed us to identify context-specific signaling through joint manifold learning and quantitative comparisons of multiple cell–cell communication networks [12]. Our analysis was based on a consensus ligand-receptor (LR) dataset by Dimitrov et al. [13]. The consensus LR dataset contained 4701 LR pairs compiled from 16 intercellular communication inference resources [13].

We followed the comparison analysis pipeline of CellChat to identify dysregulated intercellular signaling in AD and controls. The comparison analysis requires the same cell type compositions between two conditions. We performed a compositional analysis of single-cell data analysis using scCODA [26]. We found no significant difference in cell type compositions between the AD and control samples in either the discovery or the replication dataset (Additional file 1: Fig. S1e, f). Then, the intercellular communication probability of each LR pair between two cell populations within each condition (AD and control) was separately modeled and calculated. This calculation integrated the ensemble average gene expression per cell group and the consensus LR dataset, using the *computeCommunProb* function using the default parameters (type = “trimean”). Permutation tests were used to recognize statistically significant intercellular communications. To identify up- and downregulated signaling LR pairs in AD, we used the *netVisual_bubble* function with the default parameters. An LR interaction between two cell types was considered to be context-specific if it had permutation test *p*-value < 0.05 under the corresponding condition and it exhibited different communication probabilities compared to the alternate condition. Further filtering of identified interactions was based on the differential gene expression profile (between AD and controls) of sender and receiver cell types. Interactions were retained only if (1) the ligand and receptor genes were expressed in more than 10% of sender and receiver cells and (2) the gene expression of ligands met an absolute log2 fold change > 0.1 with an adjusted *p*-value < 0.05.

### Intracellular target genes analysis of dysregulated intercellular communication signals

We extended our analysis by applying the NicheNet tool [27] to assess the regulatory potential of dysregulated intercellular signals, as identified in CellChat comparison analysis, on their potential target genes within the receiver cells. For each sender-receiver cell type analysis, we focused on the ligands involved in the dysregulated LR interactions identified in our previous CellChat comparison analysis. Additionally, we considered differentially expressed genes (DEGs) in AD versus controls within each receiver cell population as the potential target genes of interest for the NicheNet analysis. Furthermore, we utilized a ligand-to-target model from NicheNet [27], which encapsulated prior knowledge regarding the regulatory potential between specific ligands and their impact on the expression of target genes.

We identified the DEGs in each receiver cell population using the *FindMarkers* function in the Seurat package with default parameters in the discovery and replication datasets separately. Additionally, sex, age of death, and number of features were included as covariates in the differential analysis. The DEGs were filtered based on the Bonferroni adjusted *p*-value < 0.05 using all genes in the datasets [28].

Following the NicheNet pipeline, we used the *predict_ligand_activities* function to calculate the ligand activity, which is based on the enrichment of their predicted target genes in the set of genes that are differentially expressed in the receiver cell. The area under the precision-recall curve (AUPR) was calculated and used to prioritize the ligands. Regulatory potential scores were then calculated between prioritized ligands and potential targeted genes within receiver cells using the default parameters. Active ligand-target connections were visualized if their regulatory potential score exceeded the 25% quantile of scores of interactions between prioritized ligands and their top targets. Genes expressed in over 10% of receiver cells were considered background genes.

In addition, we utilized the ClusterProfiler R package (version 4.2.2) for over-representation analysis to find enriched Gene Ontology (GO) Biological Processes (BP) [29]. Specifically, the identified genes in the dysregulated LR interaction in specific sender and receiver cell types, as well as the inferred intracellular target genes, were used as the query gene list. In the context of the cell type-specific analysis, we defined our background gene set to encompass all ligand and receptor genes from the consensus LR dataset that were expressed in the relevant cell types, as well as genes that were expressed in either the sender or receiver cells. We characterized “expressed genes” as those that were observed in more than 10% of cells in either the sender or receiver cell types. The enriched terms were filtered using a Benjamini–Hochberg adjusted *p*-value < 0.05 [30].

### Gene Ontology (GO) terms filtration

To examine the biological relevance of dysregulated intercellular signals in AD, we conducted pathway enrichment analysis of the genes in the dysregulated LR interactions identified in the intercellular communication analyses in both discovery and replication datasets. We used 10,532 GO terms from three domains, including biological processes (BP), molecular functions (MF), and cellular components (CC), from the Molecular Signatures Database (version 2023.1.Hs, accessed on March 6th, 2023) [31]. We limited the GO terms to 25 to 500 genes to filter small or large gene sets. Only the GO terms containing at least one dysregulated LR gene pair, including both ligand and receptor genes, as identified in the preceding analytical step, were retained as the candidate GO terms for further investigation.

### Pathway enrichment analysis using GWAS summary statistics

We implemented the GWAS statistic fine-mapping tool, MAGMA, to detect AD-associated pathways encompassing the replicated dysregulated LR interactions (Fig. 1b) [32]. In the past decade, more than six AD GWASs were published [18–21, 33, 34]. These studies included shared and distinct participants, allowing for the characterization of new genetic risk factors associated with AD. In our analysis, we used the summary statistic data from a meta-analysis GWAS performed by Wightman et al. [20], including 398,058 individuals (39,918 clinically diagnosed AD cases and 358,140 controls) of European descent, with proxy cases from the UK BioBank and 23andMe excluded [20].

Following the standard pipeline, we first employed the MAGMA tool to evaluate gene-level significance using the collected AD GWAS summary statistics [20]. A gene annotation with a 35 kb window upstream and a 10 kb window downstream was used for the MAGMA gene analyses. Subsequently, we conducted the pathway analysis employing MAGMA to identify AD-associated GO terms containing replicated dysregulated LR interactions [32]. The final results were filtered using a Benjamini–Hochberg adjusted *p*-value < 0.05 [30].

### Pathway-based polygenic scores analysis using WGS data

We utilized PRSet, a recently released pathway-based polygenic scores (PGSs) analysis tool, to further evaluate the potential association of GO terms with AD, focusing on those terms encompassing replicated dysregulated LR interactions [35]. Briefly, the PRSet method employs a classical clumping + thresholding (C + T) technique to calculate pathway-specific PGSs in relation to selected GO terms for individuals with genotyping data. Single-nucleotide polymorphisms (SNPs) falling within regions of interest are preferentially retained for each linkage disequilibrium clump of SNPs, with a clumping distance of 500 kb to either side of the index SNP and an LD *r*^2^ threshold > 0.2.

The identical AD GWAS summary statistics from the MAGMA analysis and the gene coordinates of each gene were used as the base data in the PRSet analysis [20]. In addition, a WGS dataset of 1894 individuals of AD individuals and controls, downloaded from the AD Knowledge Portal, was used for pathway-specific PGSs evaluation [24, 36]. The WGS dataset includes participants from three large cohorts: 1200 individuals from ROSMAP [37], 354 from the Mount Sinai Brain Bank [38], and 350 from the Mayo Clinic [39]. The original WGS data were aligned to the human reference GRCh37 and processed using the GATK best practices workflow [36]. The dataset was subsequently refined based on race, resulting in 1746 individuals of European descent for the analysis.

The performance of the generated PGS for each GO term was initially determined through a generalized linear model. The covariates used in the model were sex, age at death, the number of *APOE4* alleles, and the first ten principal components. We used PRSet competitive *p*-values calculation, based on permutation test, to assess signal enrichment compared to identically clumped SNPs in regions of the genome considered background (all genes). A pathway PGS with competitive *p*-values ≤ 0.05 was considered significantly enriched in AD.

### Drug repurposing analysis 

Based on the drug target analysis strategies from our previous work [40], we identified drugs that could potentially be repurposed to target genes involved in dysregulated LR interactions in AD. Here, we employed the Therapeutic Target Database to obtain information on drugs and their corresponding investigational, literature-curated, and FDA-approved targets [41]. We then prioritized candidate repurposing drugs and compounds based on their ability to cross the blood–brain barrier (BBB) based on existing literature [41, 42].

## Results

### Identification of dysregulated intercellular networks in AD

The integrative analysis framework is depicted in Fig. 1. To systematically examine the intercellular communication signals in healthy controls and AD individuals, we analyzed two snRNA-seq datasets of postmortem PFC samples [9, 11], utilizing one for discovery and the other for replication, as stated in the “Methods” section. The discovery dataset was derived from postmortem PFC samples of 24 AD participants and 24 age of death and sex-matched controls from the ROSMAP cohort [9]. The replication dataset was derived from postmortem PFC tissue from 11 AD participants and seven age of death-matched controls from UCI MIND-ADRC [11]. After performing universal preprocessing and quality control procedures, we analyzed 69,499 nuclei from the discovery dataset and 56,440 nuclei from the replication dataset, corresponding to six major brain cell types, namely astrocytes, excitatory and inhibitory neurons, microglia, oligodendrocytes, and OPCs (Additional file 1: Fig. S1). Using CellChat v.1.6.0 [12], we identified intercellular communication signals based on a LR dataset containing 4701 consensus LR pairs compiled from 16 intercellular communication inference resources [13]. Our analysis followed the standard comparison analysis pipeline, wherein we separately inferred intercellular communication signals for AD and control samples. Subsequently, we conducted an integrative comparison analysis to discern the differences in cell–cell communication signals between the two conditions.

**Fig. 2:**
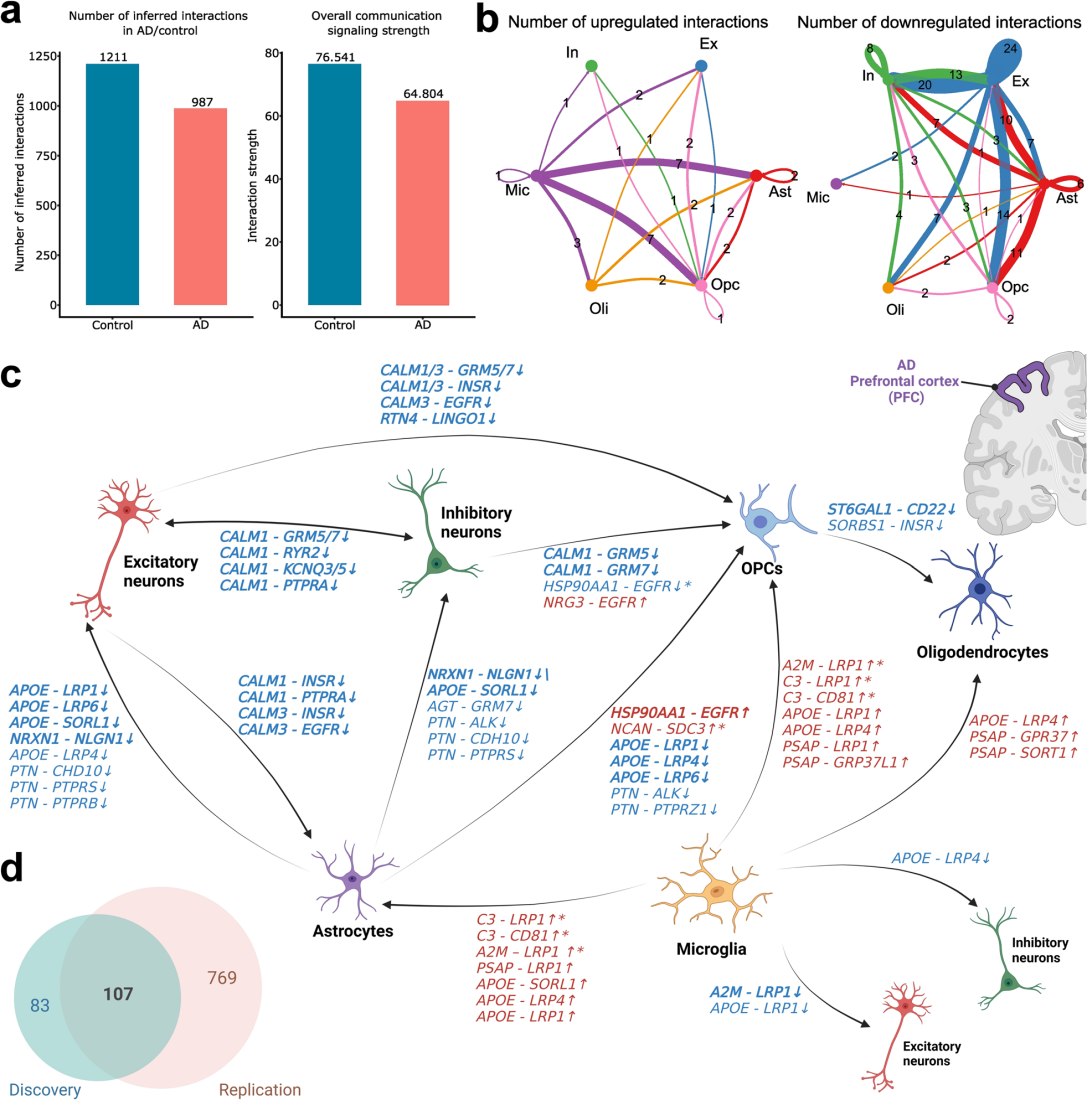
Comparative analysis of cell–cell communication signals between Alzheimer’s disease (AD) and controls. **a** The total number of inferred interaction signals and total interaction strength in AD (red) and controls (blue). **b** Number of inferred upregulated and downregulated interaction signals across cell types in Alzheimer’s disease (AD) and controls in the discovery dataset. **c** Highlighted dysregulated ligand-receptor (LR) pairs across major cell types in AD. The bolded LR pairs were replicated in the independent replication dataset using the identical analysis workflow. The stared LR pairs were identified with different communication probabilities in the independent replication dataset, but with opposite directions. **d** Venn diagram of the number of dysregulated LR pairs identified in the discovery and replication datasets. Ast astrocyte, Ex excitatory neuron, In inhibitory neuron, Mic microglia, Oli oligodendrocyte, OPC oligodendrocyte precursor cell

In the discovery dataset, we identified 987 and 1211 LR interactions (permutation *p*-value < 0.05) across cell type pairs in AD and controls (Fig. 2a). Among these LR interactions, 934 LR interaction pairs were consistently inferred in both AD and control samples, as depicted in Additional file 1: Fig. S2a. Notably, we found 53 LR interactions that were significantly inferred solely in the AD samples and an additional 277 LR interactions that exhibited exclusive significance in the control samples within the discovery dataset. For instance, we observed the LR interaction *A2M-LRP1*, which originated from microglia and targeted astrocytes, to be exclusively significant in the AD group, as indicated by a permutation *p*-value < 0.05. Moreover, we found a decrease in interaction strength within the AD group (64.804) versus in controls (76.541), which was computed by summing the communication probabilities of all inferred LR pairs in each condition (Fig. 2a). This suggests a general decline in intercellular communication in AD. Focusing on cell-type-specific communication alterations, we found that both outgoing and incoming intercellular communication signals in excitatory and inhibitory neurons exhibited decreases in both quantity and strength in AD samples (Additional file 1: Fig. S2b). In non-neuronal cell types, astrocytes, microglia, and OPCs showed decreased incoming and outgoing communications connecting with neuronal cell types (Additional file 1: Fig. S2b).

### Non-neuronal cell type mediated dysregulation in LR interactions revealed neuroinflammation and calcium dyshomeostasis in AD

Our investigation delved into the alterations in each LR gene pair, aiming to find the dysregulated LR interactions that may be driving the intercellular communication disruption in AD. In total, we identified 190 dysregulated LR interactions across six major brain cell types (Fig. 2b, c, Additional file 2: Table S1), defined as the LR interactions (permutation *p*-value < 0.05) that exhibited different communication probabilities in AD and had genes encoding ligands differentially expressed in AD (absolute log2 fold change > 0.1 with an adjusted *p*-value < 0.05, Additional file 2: Table S1). Among them, 38 LR interactions were upregulated and 152 were downregulated in AD (Fig. 2b). Our analysis of the discovery dataset revealed complex intercellular communication patterns across various sender and receiver cell types. Notable interactions occurred in the astrocytes-to-neurons, between excitatory and inhibitory neurons, and in the microglia-to-astrocytes signaling pathways.

In astrocytes-to-neurons signaling, we found majorly decreased intercellular signals from astrocytes to the two major neuronal cell types (Fig. 2b, c). The dysregulated LR interactions involve known AD risk genes as ligands or receptors, such as *APOE-LRP1* and *APOE-SORL1*. The *APOE-LRP1* interaction is known to mediate the clearance of β-amyloid across the BBB, thereby regulating β-amyloid transcytosis from the brain to the periphery. Targeting this pair has been suggested as a potential AD treatment [43, 44]. The receptor encoded by *SORL1* has been implicated in β-amyloid clearance [45]. In addition to pinpointing well-known AD-associated intercellular signals, our analysis identified potential novel downregulated LR interactions in AD, such as *PTN-PTPRS* and *PTN-CHD10*. Pleiotrophin (PTN) is a heparin-binding growth factor that regulates peripheral and central immune responses. The interaction of PTN and PTPRS has been reported to play a role in neuroinflammation, an important component in AD [46]. In addition, *NRXN1*, encoding a presynaptic cell adhesion molecule that interacts with Neuroligin 1 (NLGN1), was downregulated in our analyses. Notably, NLGN1, which modulates the toxicity of β-amyloid oligomers, was observed to be altered in the hippocampus of AD individuals [47–50].

Furthermore, we detected downregulation in the communication signaling between excitatory and inhibitory neurons (Fig. 2c, Additional file 1: Fig. S2d). The implicated genes encoding ligands calmodulin (CALM), specifically *CALM1* and *CALM3*, were present in 18 downregulated LR interactions between excitatory neurons and inhibitory neurons. Our analysis also revealed downregulated *CALM* signals from neurons to non-neuronal cell types, such as astrocytes and OPCs. This finding indicates a potential association between calcium ion channel dysfunction, calcium dyshomeostasis, and AD pathology [51].

Remarkably, several upregulated LR interactions from microglia to astrocytes were identified, including *C3-CD81* and LR interactions with ligands encoded by *APOE* and *PSAP.* Particularly, complement component 3 (C3) and its receptor CD81 molecule (CD81) are recognized for their neuroinflammatory function between microglia and astrocytes, suggesting potential implications in AD pathophysiology (Fig. 2c, Additional file 1: Fig. S2c) [52, 53].

### Replication analysis of dysregulated intercellular communication in AD

To increase the validity of the results, we replicated the analysis using an independent snRNA-seq dataset (*n* = 56,440 nuclei) from UCI MIND-ADRC [11]. In the replication dataset, we identified 876 dysregulated LR interactions (258 upregulated and 618 downregulated LR interactions) across six major brain cell types (Additional file 3: Table S2). As shown in Fig. 2d, we successfully replicated 107 out of 190 dysregulated LR interactions across the six major cell types (Additional file 4: Table S3). Specifically, we found consistent downregulation in LR interactions in astrocytes-to-neurons and between excitatory and inhibitory neurons signaling. However, in the microglia-to-astrocytes signaling, we found LR interactions, including *C3-LRP1*, *C3-CD81*, and *A2M-LRP1*, were upregulated in AD in the discovery dataset but downregulated in the replication dataset (Fig. 2c). On the other hand, the LR interaction, *HSP90AA1-EGFR* showed consistent upregulation from the astrocytes to OPC. The EGFR signaling pathway is known to be associated with AD, and recent studies suggest that EGFR inhibitors can have potential beneficial effects in mitigating pathological sequelae in AD [54].

### Dysregulated intercellular signaling pathways

Our comparative analysis identified dynamic communication patterns at the signaling pathway level (Additional file 1: Fig. S2e-g) in the discovery dataset, revealing distinct changes in information flow between AD and control subjects. Interestingly, the two well-known AD-associated pathways, APOE and PSAP signaling pathways, demonstrated a similar flow of information between conditions, albeit with a reduced signaling strength among astrocytes (Additional file 1: Fig. S2e). Moreover, most identified pathways exhibited decreased signaling strength in AD across various cell types. Notably, the outgoing and incoming signaling strengths of the IGF pathway were downregulated in AD in astrocytes, excitatory and inhibitory neurons (Additional file 1: Fig. S2f, g). This suggests a need for further examination of individual LR pairs within each signaling pathway.

### Intracellular communication analysis revealed the regulatory potential of dysregulated ligand genes on the target genes in AD

**Fig. 3:**
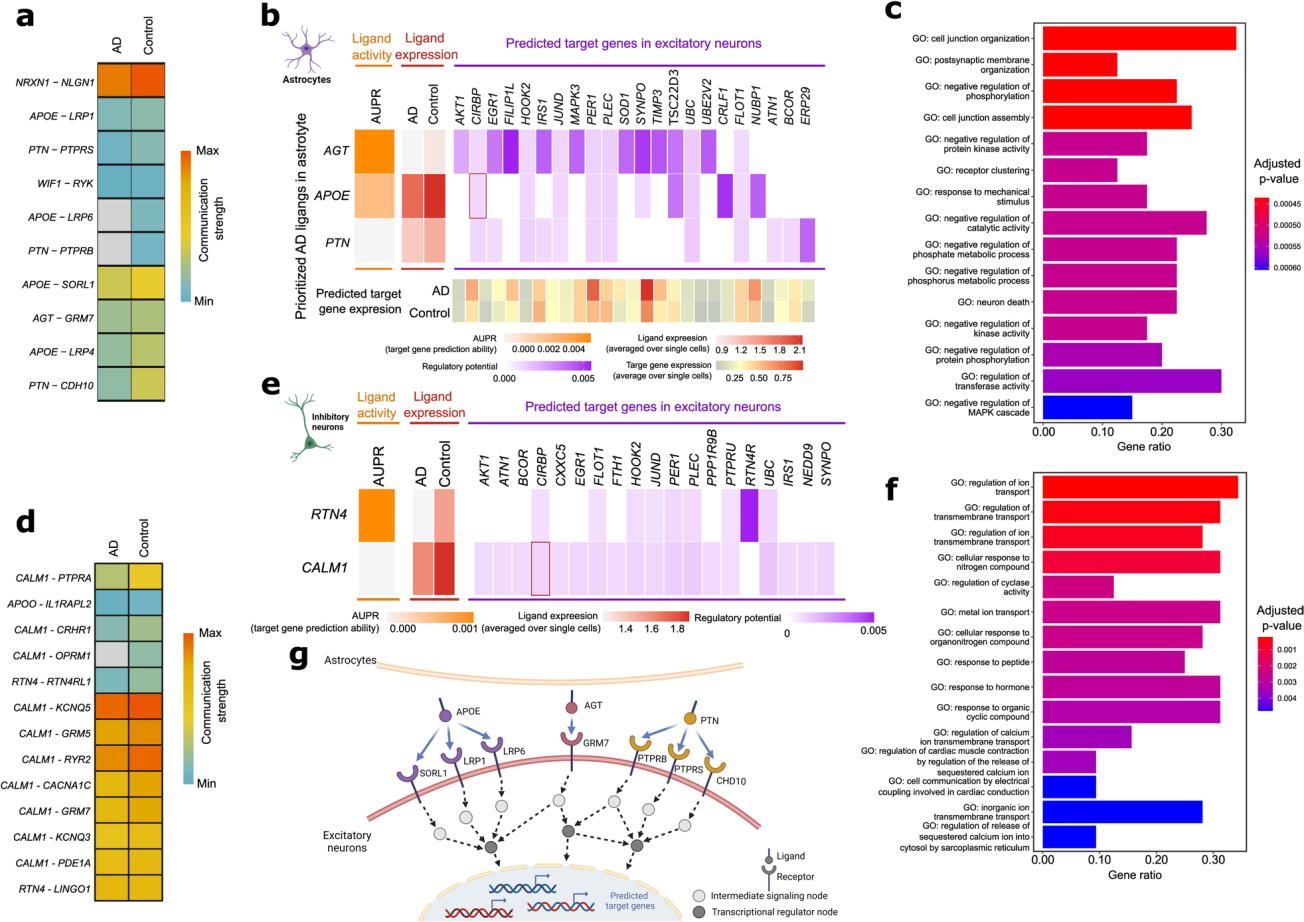
Ligand–target gene analysis of dysregulated ligand-receptor (LR) interactions across astrocyte, inhibitory, and excitatory neurons. **a** Communication strength of dysregulated LR pairs from astrocytes to excitatory neurons. **b** The heatmap depicts the regulatory potential scores (purple) of each ligand of dysregulated LR pairs in the sender cell (astrocytes) to differentially expressed genes (DEGs) in the receiver cell (excitatory neurons). Ligands were ranked by the area under the precision-recall curve (AUPR, orange) and level of expression in astrocytes (red). The expression level of the predicted target gene in excitatory neurons is shown (yellow to red). **c** Bar plot shows the top 15 Gene Ontology Biological Processes (GO BP) significantly enriched by genes involved in dysregulated LR pairs between astrocytes and excitatory neurons and predicted target genes. **d** Communication strength of dysregulated LR pairs from inhibitory to excitatory neurons. **e** The heatmap depicts the regulatory potential scores (purple) of each ligand of dysregulated LR pairs in inhibitory neurons to DEGs in excitatory neurons. Ligands were ranked by the AUPR (orange) and level of expression in astrocytes (red). **f** Bar plot shows the top 15 GO BP significantly enriched in dysregulated LR pairs between inhibitory and excitatory neurons and predicted target genes. **g** Schematic figure shows the dysregulated LR pairs between astrocyte and excitatory neurons and predicted target genes

Using NicheNet ligand activity analysis, we assessed the regulatory potential of ligand genes involved in dysregulated LR interactions, previously identified in our CellChat comparison analysis, on the DEGs within the receiver cells (Fig. 3, Additional file 1: Fig. S3). This analysis incorporated prior ligand-target regulatory relationships, as provided by the NicheNet package, revealing the influence of these ligand genes on DEGs in the receiver cells.

Ligand genes of downregulated LR pairs from astrocytes to excitatory neurons, such as *AGT*, *APOE*, and *PTN*, exhibited increased ligand activity but relatively low expression in AD (Fig. 3a, b, g). Notably, angiotensinogen,﻿ encoded by *AGT,* displayed the most substantial potential regulatory influence on DEGs in excitatory neurons, including *FILIPL1*, *SYNPO*, and *MAPK3* (Fig. 3b). Additionally, ligand-target gene pair, *APOE* linked to *CIRBP*, was replicated in the replication dataset. We performed an over-representation analysis on genes involved in dysregulated LR interactions and predicted target genes to further elucidate underlying enriched GO BP. The background gene set for this analysis included all genes from the consensus LR dataset that were expressed in the relevant cell types and genes expressed in either astrocytes or excitatory neurons, as described in the “Methods” section. The results indicated significant enrichment in the cell junction organization, postsynaptic membrane organization, and negative regulation of phosphorylation, among others (adjusted *p*-values < 0.05, Fig. 3c). The schematic information flow was depicted in Fig. 3g.

We next explored the regulatory potential of dysregulated ligand genes in inhibitory neurons on the DEGs of excitatory neurons. Ligands encoded by genes, such as *RTN4* and *CALM1*, exhibited high ligand activity, potentially modulating the predicted target genes in excitatory neurons (Fig. 3d, e). Over-representation analysis of dysregulated LR gene pairs and predicted target genes between inhibitory and excitatory neurons yielded significantly enriched GO BPs. The top enriched GO BPs included regulation of ion transport-related functions, cellular response to nitrogen compound, and regulation of cyclase activity, among others (adjusted *p*-values < 0.05, Fig. 3f). These findings suggest a critical role of transmembrane signaling dysregulation in AD pathology.

### Integrative pathway-level analyses revealed AD-associated GO terms encompassing dysregulated LR interactions

Our comparative intercellular communication analyses identified 107 dysregulated LR interactions across six major brain cell types in the discovery and replication datasets (Fig. 2, Additional file 4: Table S3). To gain a more profound understanding of the biological relevance of dysregulated intercellular signaling in AD, we performed pathway analyses by incorporating AD GWAS summary statistics and WGS data (Fig. 1b). We downloaded all GO terms from three domains (BP, MF, and CC) from Molecular Signatures Database (version 2023.1.Hs, accessed on March 6, 2023) [31]. We found 229 GO terms containing at least one dysregulated LR gene pair (both ligand and receptor genes) that were identified and replicated in comparative intercellular communication analyses in discovery and replication snRNA-seq datasets.

**Fig. 4:**
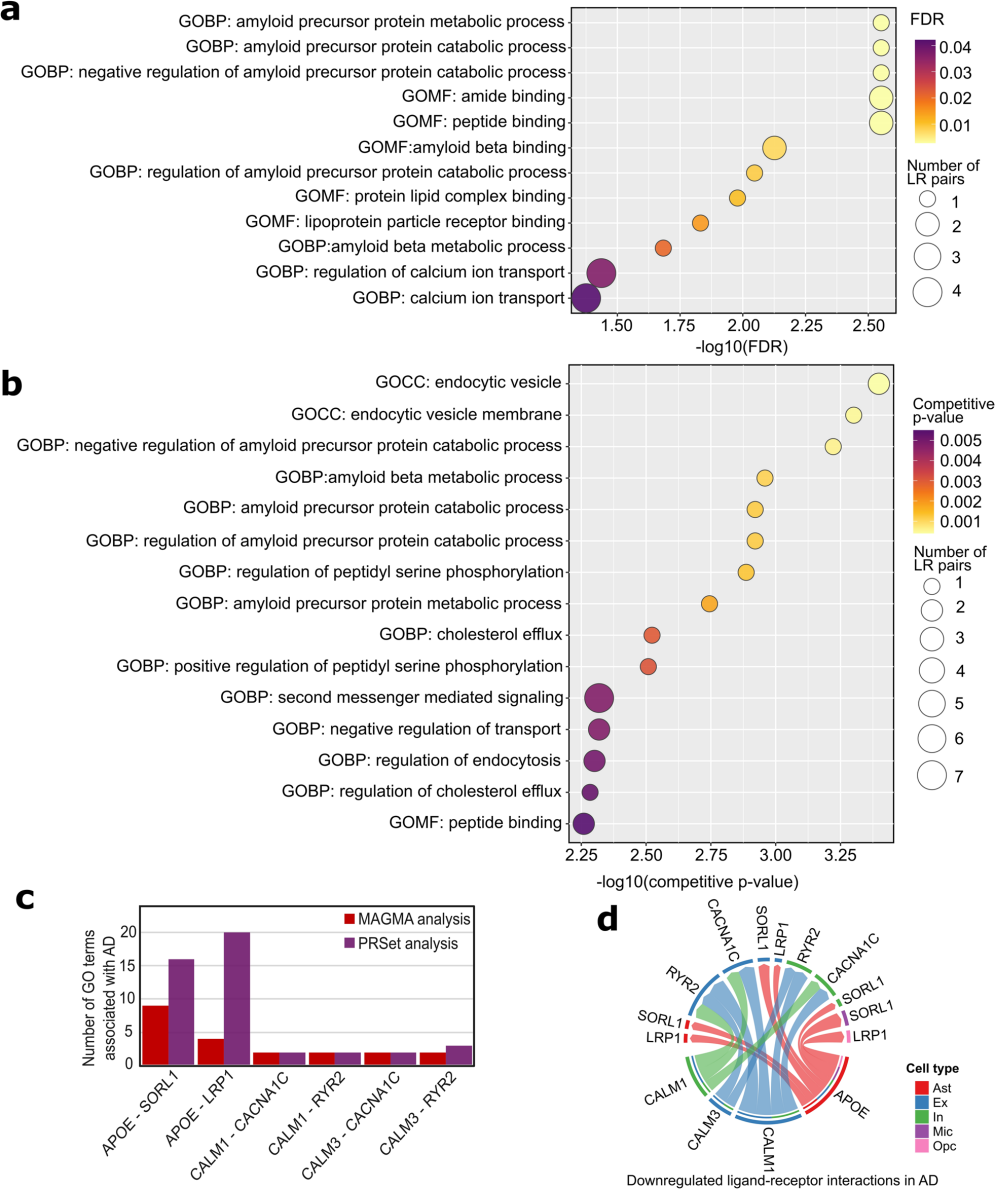
Identification of Alzheimer’s disease (AD)-associated Gene Ontology (GO) terms encompassing the replicated cell type-specific dysregulated ligand-receptor (LR) pairs through **a** MAGMA or **b** PRSet analyses. The dot size represents the number of dysregulated LR pairs included in the pathway. **c** Bar plot shows the dysregulated LR pairs that were validated in both MAGMA and PRSet analyses. **d** Circle plot shows the downregulated intercellular signaling of six prioritized LR pairs between involved cell types. Ast astrocyte, Ex excitatory neuron, In inhibitory neuron, Mic microglia, OPC oligodendrocyte precursor cell

We first performed MAGMA pathway analysis to leverage AD GWAS summary statistics [20] on 229 candidate GO terms that contain at least one replicated dysregulated LR interaction pair. We found a total of 12 GO (7 BP and 5 MF) terms significantly associated with AD (adjusted *p*-values < 0.05, Fig. 4a, Additional file 5: Table S4). The most significant GO BP was related to “amyloid precursor protein metabolic and catabolic processes” (Fig. 4a, adjusted *p*-value < 0.01). Furthermore, the MAGMA pathway analysis highlighted several GO MF terms, including “amide binding,” “peptide binding,” and “amyloid-beta binding” (Fig. 4a, adjusted *p*-values < 0.01). The LR gene pair *APOE-SORL1* was consistently present across most of the significant GO terms identified by MAGMA. Interestingly, regulation of calcium ion transport, harboring *CALM1* and *CALM3*-related dysregulated LR pairs, was identified as significantly associated with AD. In total, six LR interactions were validated by MAGMA (Additional file 5: Table S4).

Pathway-level PGSs have been suggested to better inform disease biology than classical PGSs [35]; therefore, we performed a pathway-level PGS analysis utilizing the PRSet tool on candidate GO terms containing dysregulated LR interactions [35]. We used the same AD GWAS summary statistics [20] as the base for PGS calculation and then the WGS data of 1746 individuals of European descent from three AD cohorts [37–39] to evaluate PGS performance, as described in the “Methods” section. Our analysis revealed the PGSs of 44 GO terms significantly associated with AD (competitive *p*-values < 0.05, Additional file 6: Table S5). Figure 4b shows the top 15 GO terms associated with AD, primarily centered on endocytosis-related cellular components, such as endocytic vesicle and endocytic vesicle membrane. Additionally, the PRSet analysis indicated significant enrichment of “amyloid precursor protein catabolic and metabolic processes” in AD.

We identified 17 replicated LR interactions involved in GO terms significantly associated with AD supported by either MAGMA or pathway-level PGS analysis (Additional file 7: Table S6). Among these replicated LR interactions, six were intricately intertwined with GO terms highlighted by both analyses. These six LR interactions, namely *APOE-SORL1, APOE-LRP1, CALM1-CACNA1C, CALM1-RYR2, CALM3-CACNA1C,* and *CALM3-RYR2* (Fig. 4c, d), consistently exhibited downregulation patterns in AD in comparative intercellular communication analysis. We observed that *APOE-SORL1* and *APOE-LRP1* involved downregulation in signaling, sending from astrocytes, and targeting neurons and other non-neuronal cells in the discovery and the replication datasets (Additional file 4: Table S3). Furthermore, the intercellular signaling originating from neuronal cells and directed towards other non-neuronal cell types, mediated by the ligands encoded by *CALM1* and *CALM3* and their corresponding receptors encoded by *CACNA1C* and *RYR2*, displayed a downregulated trend in both the discovery and replication datasets (Additional file 2: Table S1 and Additional file 3: Table S2).

### Prioritization of repurposable drug targeting dysregulated cell–cell communication signals in AD

Finally, following our previous work, we explored potential repurposable drugs targeting the 17 high-confident dysregulated LR pairs [40]. By inquiring the Therapeutic Target Database, we identified 9 FDA-approved drugs capable of crossing the BBB [41, 42]. These drugs target one ligand (HSP90AA1) and three receptors (EGFR, CACNA1C, INSR) within the dysregulated LR interactions (Table 1). Additionally, four receptors in these pairs were found to be targets of either investigational drugs or previously reported drugs in the literature (Table 1), including GRM5, GRM7, LRP1, and APOE. GRM5 refers to the metabotropic glutamate receptor 5 (mGluR5), targeted by the drug ADX-48621, which is currently being investigated for Parkinson’s disease, dyskinesia, and mood disorders (https://clinicaltrials.gov/, NCT04857359). GRM7 refers to the metabotropic glutamate receptor 7 (mGluR7) and is targeted by the drug MPPG, which is still a discovery agent [55]. Currently, there is no drug targeting LRP1 under investigation. For APOE, the drug AEM-28 is under study for hyperlipidemia [41].

**Table 1:** Drug repurposing analysis of Therapeutic Target Database

Target gene	Target name	Ligand-receptor gene pair	Approved repurposable drug	Indication
*CACNA1C*	Voltage-gated calcium channel alpha Cav1.2 (CACNA1C)	*CALM1-CACNA1C, CALM-CACNA1C*	Rauwolfia serpentina root	Discovery agent
*EGFR*	Epidermal growth factor receptor (EGFR)	*HSP90AA1-EGFR, CALM3-EGFR*	Erlotinib	Non-small-cell lung cancer
			Gefitinib	Solid tumor/cancer
			Osimertinib	Non-small-cell lung cancer
*HSP90AA1*	Heat shock protein 90 alpha (HSP90A)	*HSP90AA1-EGFR*	Amlexanox	Respiratory tract inflammation
			Cromoglicate	Respiratory tract inflammation
*INSR*	Insulin receptor (INSR)	*SORBS1-INSR*	Insulin analogues	Diabetic complication
			Metformin arginine-hemisuccinimide	Type-2 diabetes
			Ryzodeq	Type-2 diabetes
*GRM5*	Metabotropic glutamate receptor 5 (mGluR5)	*CALM1-GRM5, CALM3-GRM5*	NA	Clinical trial target
*GRM7*	Metabotropic glutamate receptor 7 (mGluR7)	*CALM1-GRM7, CALM3-GRM7*	NA	Literature-reported target
*LRP1*	Apolipoprotein E receptor (LRP1)	*APOE-LRP1*	NA	Literature-reported target
*APOE*	Apolipoprotein E (APOE)	*APOE-SORL1*	NA	Clinical trial target

## Discussion

In this study, we integrated human brain snRNA-seq datasets, GWAS summary statistics, and WGS from AD and control individuals to identify cell type-specific dysregulated LR pairs and their underlying biological pathways. We identified key known and potential novel dysregulated LR interactions and highlighted vulnerable cell types in AD. Our pathway analyses further prioritized dysregulated LR interactions and related biological pathways supported by genetic association data. Our analysis provides a detailed landscape of cellular communication alterations in AD (Figs. 2 and 4), highlighting the power of multi-layered data integration in the study of complex diseases.

Our integrative analysis revealed the critical role of dysregulated astrocytes-to-neurons signaling and related biological functions associated with AD. Our comprehensive bioinformatics analysis highlights that the well-known gene *APOE*, which encodes the ligand in three dysregulated LR pairs, interacts with receptors encoded by *LRP1*, *LRP6*, and *SORL1*. The three dysregulated LR pairs were identified as downregulated in AD compared to controls in discovery and replication datasets (Fig. 2b, c). In addition, LR pairs containing *APOE* (*APOE-LRP1* and *APOE-SORL1*) were found to be involved in the top GO terms significantly associated with AD in our pathway analyses, such as “GO CC: endocytic vesicle” and “GO BP: regulation of amyloid precursor protein catabolic process” (Fig. 4a, b). Our integrative analysis, considering single-nucleus transcriptome and genotyping data of AD and controls, underscored the significant role of *APOE* signaling in the interplay between non-neurons and neurons in the pathophysiology of AD [27, 28]. In addition, pleiotrophin, encoded by *PTN*, is a heparin-binding growth factor that regulates peripheral and central immune responses. In the discovery dataset, we found that PTN-involved LR interactions (PTN-PTPRS and PTN-PTPRB) were downregulated from astrocytes to excitatory and inhibitory neurons. The interactions of PTN with protein tyrosine phosphatase receptor type Z polypeptide 1 (PTPRZ1) and protein tyrosine phosphatase receptor type S polypeptide (PTPRB) may play a role in cell proliferation and regulation, both of which are important in AD [46].

Our analyses underscore the pivotal role of calcium dyshomeostasis in the pathogenesis of AD. Notably, CALM, encoded by *CALM1* and *CALM3*, serves as a ligand in 18 replicated downregulated LR pairs between excitatory and inhibitory neurons in AD. These LR pairs displayed alterations between excitatory and inhibitory neurons in our analysis. Among them, ten LR gene pairs (*CALM1-GRM5, CALM1-GRM7, CALM1-RYR2, CALM3 -GRM3,* and *CALM5-GRM7, CALM3-RYR2, CALM1-CACNA1C, CALM3-CACNA1C, CALM3-EGFR, CALM3-INSR*) were prioritized in the pathway analyses (Additional file 7: Table S6). Interestingly, the metabotropic glutamate receptor (GRM) was found to be the receptor in five of these 18 LR pairs. In general, CALMs interact with GRMs to regulate synaptic plasticity. *GRM5* gene is ubiquitously expressed in brain regions implicated in AD phenotypes in mice and in regions linked to memory and learning [56, 57]. Our pathway analyses highlighted GO BPs—such as regulation of calcium ion transport, second messenger-mediated signaling, and maintenance of location, which encompass four dysregulated LR pairs, including *CALM1-RYR2, CALM3-RYR2, CALM1-CACNA1C,* and *CALM3-CACNA1C* (Fig. 4, Additional file 7: Table S6). RYR2 is a receptor to CALM1, and the binding of CALM1 to RYR2 has been shown to limit neuronal loss in AD [58]. Voltage-dependent L-type calcium channel subunit alpha-1C (CACNA1C) interacts with CALM1 and CALM3 to regulate calcium influx. It can be related to neuronal survival and synaptic efficiency and is thought to be involved in attention, learning, memory, and stress response [59–62].

Our ligand-target gene analysis revealed the potential regulatory role of ligand encoded by *CALM1* on the DEGs in excitatory neurons. The predicted target gene, *CIRBP*, was replicated in the independent dataset (Fig. 3e). Cold-inducible RNA-binding protein (CIRBP) is a general stress-response protein, which was downregulated in AD in our analysis (Fig. 3e). It has been proposed that CRIBP exerts a protective effect against neuronal amyloid toxicity via antioxidative and antiapoptotic pathways [63].

In our analysis, most intercellular signals mediated by LR pairs were downregulated across six major cell types in AD. Notably, we observed LR that interactions from microglia to astrocytes were upregulated in the discovery dataset, although downregulated in the replication dataset (Fig. 2c). Among the dysregulated LR interactions, C3 was found to be altered as a ligand in two different LR pairs, *C3-LRP1* and *C3-CD81*. Both pairs were upregulated in microglia, astrocytes, and OPCs, with microglia as the sender and astrocytes and OPCs as the receivers in the discovery dataset (Fig. 2c). C3 is a protein that is part of the complement system and part of the immune system; it co-localizes with amyloid plaques in AD. Low-density lipoprotein receptor-related protein 1 (LRP1) is a surface receptor and mediates pathways that interact with astrocytes and pericytes, the last of which is associated with the BBB. *LRP1* expression is known to decrease in endothelial cells due to normal aging and in AD. C3 interacts and can bind with low-density LRP1 to regulate immune response and participate in several cellular processes [44, 64–67]. Ligand C3 and receptor CD81 play an inhibitory role in the control of immune responses [52]. We also identified alpha-2-macroglobulin (A2M) as a ligand in the *A2M*-*LRP1* pair, which was upregulated in microglia in the discovery dataset. A2M interacts with LRP1 to regulate cholesterol metabolism and is considered a potential therapeutic target in AD [64]. Our ligand-target gene analysis from microglia to astrocytes suggests the regulatory potential of ligands encoded by *A2M* and *C3* on the DEGs in the receiver cells (Additional file 1: Fig. S3). Over-representation analysis on genes involved in dysregulated LR pairs and predicted target genes indicated significant enrichment in GO BPs, including amyloid-beta clearance and functions related to regulation of cholesterol and sterol transport (adjusted *p*-value < 0.05, Additional file 1: Fig. S3c).

Moreover, one replicated dysregulated LR interaction, *NRXN1-NLGN1*, is related to neurexins (NRXNs) and neuroligins (NLGNs), and their signaling is decreased in AD in a myriad of cell types, including astrocytes, excitatory, and inhibitory neurons. The NRXNs are cell-surface receptors that bind NLGNs, forming a crucial transsynaptic complex at brain synapses. This transsynaptic complex is vital for efficient neurotransmission and is involved in forming synaptic contacts and functional synaptic structures. Recent reports suggest that NRXNs and NLGNs undergo proteolytic processing by presenilins at synapses, a mechanism implicated in AD, suggesting a potential dysfunction in the NRXN-NLGN pathway in AD pathology [47].

Further, we observed upregulation of other LR pairs, including PSAP-LRP1 and PSAP-GPR37, in astrocytes, microglia, and OPCs in the discovery dataset (Fig. 2c, Additional file 2: Table S1). Prosaposin (PSAP) is a highly conserved glycoprotein that is a precursor of saposins; it also serves as a neurotrophic factor and a regulator of lysosomal enzymes. PSAP is known to interact with LRP1 in AD, with the interaction between PSAP and LRP1 being involved in the regulation of amyloid-beta metabolism [68]. The expression of *PSAP* and its receptor *GPR37* is upregulated in the hippocampus of individuals with AD [69–71].

Finally, other LR pairs possibly related to AD involved genes that encode receptors, such as epidermal growth factor receptor (EGFR), insulin receptor (INSR), corticotropin-releasing hormone receptor 1 (CRHR1), and adenylate cyclase-activating polypeptide type I receptor (ADCYAP1R1) (Additional file 1: Fig. S2c, d). In general, they are involved in cell proliferation and differentiation, glucose metabolism, and stress response [72]. In addition, EGFR has been identified as the receptor in two upregulated LR pairs, involving heat shock protein 90 alpha family class A member 1 (HSP90AA1) and neuregulin 3 (NRG3) as the ligands. Both are implicated in cell proliferation and differentiation; NRG3 has been implicated in cognitive impairment [73, 74]. *INSR* was also found as a gene that encodes the receptor for sorbin and SH3 domain-containing protein 1 (SORBS1), downregulated in astrocytes, excitatory neurons, inhibitory neurons, and oligodendrocytes; the *SORBS1-INSR* is known to regulate glucose metabolism. Moreover, we found that the GO BP term “regulation of cellular and carbohydrate metabolic process” encompassing *SORBS1-INSR* was associated with AD (Additional file 6: Table S5).

Our drug target analysis revealed existing and potentially novel therapeutic targets of dysregulated LR pairs in AD. Regarding EGFR, erlotinib, gefitinib, and osimertinib were found to be potential drugs for repurposing. Both erlotinib and osimertinib are used to treat lung and pancreatic cancers and can cross the BBB (Table 1). They are tyrosine kinase inhibitors that work by blocking the kinase activity of EGFR, which is involved in cell growth and survival [75]. Erlotinib and gefitinib also have antioxidant properties [76]. It has been hypothesized that both drugs may enhance axon regeneration after neurodegeneration [54]. Moreover, two drugs that target the HSP90AA1 receptor were identified, amlexanox, and cromoglicate (also called cromolyn). Both have anti-inflammatory properties, with cromolyn specifically reducing neuroinflammation. Cromolyn has been proposed as a new therapeutic target for AD [77]. Cromolyn has been shown to reduce levels of amyloid beta by promoting microglial phagocytosis [78, 79]. It also reduces the secretion of inflammatory cytokines by the microglia [80], reducing neuroinflammation in neural cells. The root of Rauwolfia serpentina, currently a discovery agent, targets the receptor CACNA1C. This compound has acetylcholinesterase (AChE) inhibitory activities, a mechanism that has been proposed to treat AD [81], and has shown neuroprotective activity.

Recently, brain insulin resistance has been found to play a role in normal memory processes, and insulin irregularities may contribute to cognitive and brain changes associated with AD [82]. Metformin and insulin target the INSR and appeared as potentially repurposable drugs in our analyses. Evidence from clinical studies has demonstrated that metformin use contributes to a lower risk of developing AD and to better cognitive performance [83]. Intranasally administered insulin is assumed to trigger improvements in synaptic plasticity, regional glucose uptake, and alleviations of AD neuropathology. Pilot clinical trials of intranasal insulin administration in individuals with mild cognitive impairment or AD indicate that acute and prolonged intranasal insulin administration can enhance memory performance [84].

While our integrative study used multiple large-scale datasets, there were several limitations. First, our inference of dysregulated LR interaction was primarily dependent on the completeness of snRNA-seq datasets, cell type annotation, and the reliability of the LR dataset. Despite employing one of the most comprehensive snRNA-seq datasets of AD and controls currently available [9], we limited our analysis to six major cell types due to a relatively low cell count of pericytes and endothelial cells. We also performed a replication analysis to ensure the reliability of the analysis. However, more complex intercellular signals could be unveiled in rare cell types or subclasses of major cell types with the employment of larger snRNA-seq datasets. Further work is anticipated by leveraging novel computational methods, such as Scriabin [85], to identify more specific signals at individual cell levels related to AD. Second, inadequate annotation of intercellular signaling pathways and intracellular regulatory networks may impede our pathway analyses of dysregulated LR pairs in AD. To address this point, we utilized comprehensive GO gene sets to evaluate the biological functions influenced by dysregulated LR signals in AD. Third, our cell–cell communication analysis was limited to the PFC region. Considering that AD pathology affects multiple brain regions, including the entorhinal cortex and hippocampus, further investigations across multiple brain regions are necessary for a more in-depth understanding of region-specific dysregulated intercellular signals in AD. Fourth, due to the cross-sectional nature of our study, we cannot ascertain causality. It is not possible to determine if alterations in the LR pairs are a cause or consequence of AD. We speculate that at least some of the altered LR pairs are causally related to AD, following the rationale that genetic variants associated with AD would affect gene expression and then protein expression of a biological pathway that involves deregulated intercellular communication in AD. Finally, rigorous laboratory experimental validation, which we did not perform because it was outside the scope of this study, would further validate the causal relationships between identified dysregulated intercellular interactions and disease progression.

## Conclusions

Our comprehensive in silico investigation provides novel insights into the complex intercellular signaling dynamics underpinning AD. By applying a novel analysis pipeline integrating snRNA-seq, GWAS, and WGS, we unveiled the intricate landscape of dysregulated LR pairs across six major cell types in AD and their potential drug targets. Notably, our findings highlight the pivotal contributions of non-neuronal cell types, particularly astrocytes, in the disruption of intercellular signaling networks in AD. These dysregulated signals, with a focus on ligands encoded by *CALM* and *APOE*, consistently emerge as key players in our comparative intercellular communication analysis, evident across both discovery and replication datasets, as well as in pathway analyses. Our discoveries lay a solid in silico foundation for further exploration of the roles of these dysregulated LR pairs in the pathogenesis of AD and their potential implications for therapeutic interventions.

### Supplementary Information

**Additional file 1:** **Figure S1.** Description of the discovery and replication datasets. The Uniform Manifold Approximation and Projection of single nuclei RNA sequencing (snRNA-seq) data of the (a) discovery dataset and (c) replication dataset after preprocessing and filtration. Major cell type proportion of each sample in (b) discovery dataset and (d) replication dataset after preprocessing and filtration. The compositional analysis of single-cell data using scCODA in the (e) discovery dataset and (f) replication dataset. Ast: astrocyte; Ex: excitatory neuron; In: inhibitory neuron; Mic: microglia; Oli: oligodendrocyte; OPC: oligodendrocyte precursor cell. **Figure S2. **(a) The Venn diagram shows the number of inferred ligand-receptor (LR) interactions and overlaps between AD and control in the discovery dataset. (b) The difference in the number of intercellular interactions and difference in interaction strength between AD and controls across major cell types in the brain. (c) Bubble plot shows the cell-type-specific upregulated LR pairs in AD. LR interactions with ligand genes differentially expressed between AD and control were highlighted and defined as dysregulated LR interactions. (d) Bubble plot shows the cell-type-specific downregulated LR pairs in AD. LR interactions with ligand genes differentially expressed between AD and control were highlighted and defined as dysregulated LR interactions. (e) The relative information flow within intercellular signaling pathways, defined as the summation of interaction strength within the pathway, quantitatively compared between AD and controls. (f) The cell type-specific outgoing signaling patterns in controls (left) and AD (right). The color represents the relative outgoing signaling strength of each signaling pathway. (g) Cell type-specific incoming signaling patterns in controls (left) and AD (right). The color represents the relative incoming signaling strength of each signaling pathway. **Figure S3.** Ligand–target gene analysis of dysregulated ligand-receptor (LR) pairs from microglia to astrocytes. (a) Communication strength of dysregulated LR pairs from microglia to astrocyte in AD and controls. (b) The heatmap depicts the regulatory potential scores (purple) of each ligand gene of dysregulated LR pairs in microglia to differentially expressed genes (DEGs) in astrocytes. The ligand genes were ranked by the area under the precision-recall curve (AUPR, orange) and the level of expression in astrocytes (red). The expression level of the predicted target gene in excitatory neurons is shown (yellow to red). (c) Bar plot shows the top 15 Gene Ontology Biological Processes significantly enriched in dysregulated LR pairs between microglia and astrocytes and their predicted target genes. (d) Volcano plot depicts the DEGs in astrocytes in the discovery dataset.

**Additional file 2:** **Table S1.** Dysregulated ligand-receptor interactions in AD identified in the discovery dataset.

**Additional file 3:** **Table S2.** Dysregulated ligand-receptor interactions in AD identified in the replication dataset.

**Additional file 4:** **Table S3.** Replicated dysregulated ligand-receptor interactions in AD identified in both discovery and replication datasets.

**Additional file 5:** **Table S4.** Alzheimer’s disease-associated Gene Ontology terms identified in the MAGMA gene-set analysis.

**Additional file 6:** **Table S5.** Alzheimer’s disease-associated Gene Ontology terms identified in the PRSet analysis.

**Additional file 7:** **Table S6.** Ligand-receptor pairs prioritized by MAGMA and PRSet pathway analyses and related Gene Ontology terms.

## Data Availability

This research is based on datasets available in online repositories. In addition, the single-nucleus transcriptome profiles of AD for supporting the findings of this study are available from the Synapse portal (https://adknowledgeportal.synapse.org/).

## Abbreviations

AD: Alzheimer’s disease
ADRC: Alzheimer’s Disease Research Center
BBB: Blood–brain barrier
BP: Biological process
CC: Cellular component
C + T: Clumping + thresholding
DEG: Differentially expressed gene
FDA: Food and Drug Administration
GO: Gene Ontology
GWAS: Genome-wide association study
LR: Ligand-receptor
MAGMA: Multi-marker Analysis of GenoMic Annotation
MF: Molecular function
OPC: Oligodendrocyte precursor cell
PC: Principal component
PFC: Prefrontal cortex
PGS: Polygenic score
ROSMAP: The Religious Orders Study and Memory and Aging Project
snRNA-seq: Single-nucleus RNA sequencing
SNP: Single-nucleotide polymorphism
UCI MIND: University of California Irvine Institute for Memory Impairments and Neurological Disorders
WGS: Whole genome sequencing
