# Supplementary material for: Unraveling the intercellular communication disruption and key pathways in Alzheimer’s disease: an integrative study of single-nucleus transcriptomes and genetic association
Source: Alzheimers Res Ther. 2024 Jan 2;16:3. doi: 10.1186/s13195-023-01372-w (PMC10762817; doi:10.1186/s13195-023-01372-w)
Supplement: Supplementary file 1 — Additional file 1: Figure S1. Description of the discovery and replication datasets. The Uniform Manifold Approximation and Projection of single nuclei RNA sequencing (snRNA-seq) data of the (a) discovery dataset and (c) replication dataset after preprocessing and filtration. Major cell type proportion of each sample in (b) discovery dataset and (d) replication dataset after preprocessing and filtration. The compositional analysis of single-cell data using scCODA in the (e) discovery dataset and (f) replication dataset. Ast: astrocyte; Ex: excitatory neuron; In: inhibitory neuron; Mic: microglia; Oli: oligodendrocyte; OPC: oligodendrocyte precursor cell. Figure S2. (a) The Venn diagram shows the number of inferred ligand-receptor (LR) interactions and overlaps between AD and control in the discovery dataset. (b) The difference in the number of intercellular interactions and difference in interaction strength between AD and controls across major cell types in the brain. (c) Bubble plot shows the cell-type-specific upregulated LR pairs in AD. LR interactions with ligand genes differentially expressed between AD and control were highlighted and defined as dysregulated LR interactions. (d) Bubble plot shows the cell-type-specific downregulated LR pairs in AD. LR interactions with ligand genes differentially expressed between AD and control were highlighted and defined as dysregulated LR interactions. (e) The relative information flow within intercellular signaling pathways, defined as the summation of interaction strength within the pathway, quantitatively compared between AD and controls. (f) The cell type-specific outgoing signaling patterns in controls (left) and AD (right). The color represents the relative outgoing signaling strength of each signaling pathway. (g) Cell type-specific incoming signaling patterns in controls (left) and AD (right). The color represents the relative incoming signaling strength of each signaling pathway. Figure S3. Ligand–target gene ana [file 13195_2023_1372_MOESM1_ESM.docx]

**Additional file 1:**


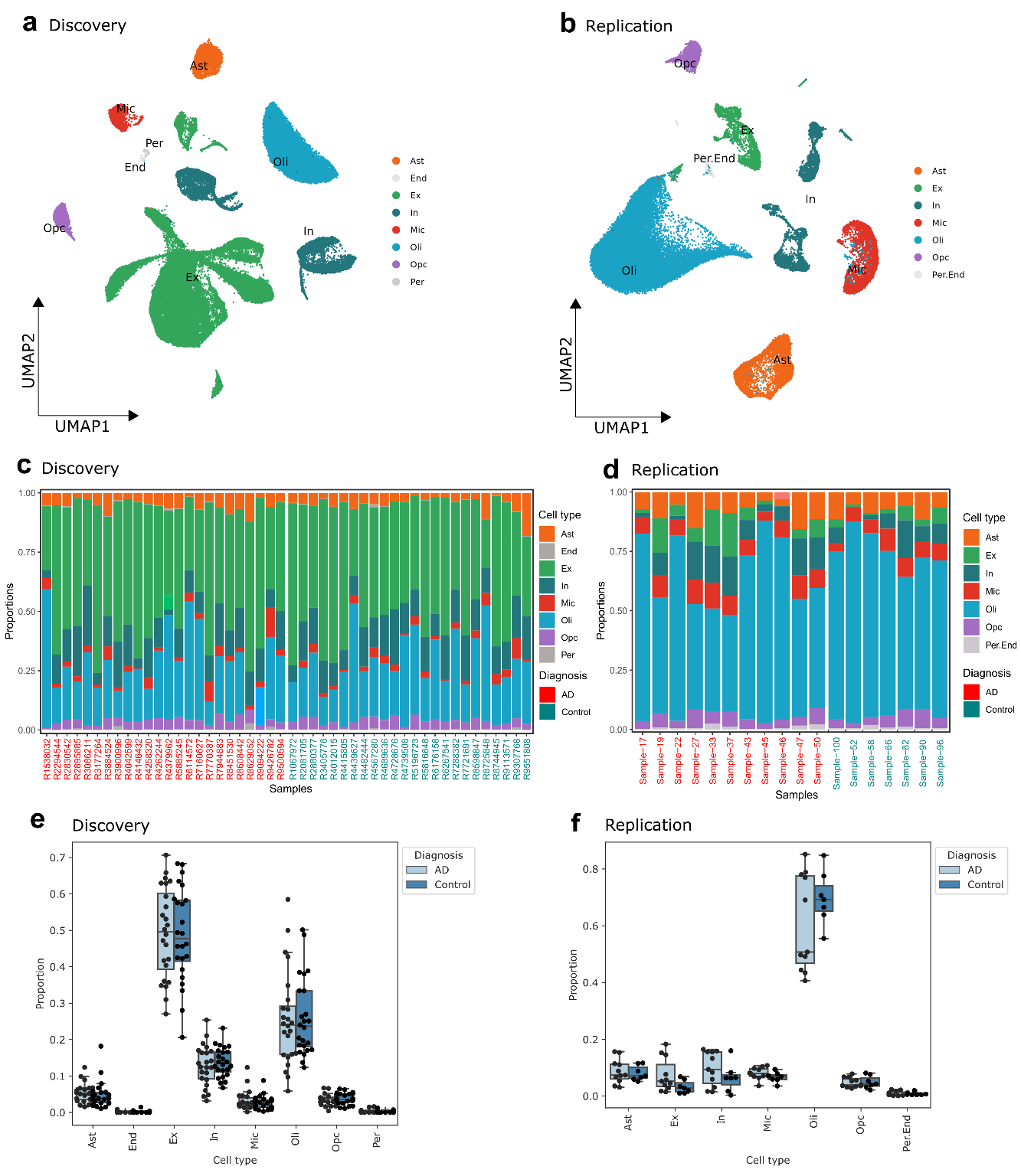


**Figure S1.** Description of the discovery and replication datasets. The Uniform Manifold Approximation and Projection of single nuclei RNA sequencing (snRNA-seq) data of the (**a**) discovery dataset and (**c**) replication dataset after preprocessing and filtration. Major cell type proportion of each sample in (**b**) discovery dataset and (**d**) replication dataset after preprocessing and filtration. The compositional analysis of single-cell data using scCODA in the (**e**) discovery dataset and (**f**) replication dataset. Ast: astrocyte; Ex: excitatory neuron; In: inhibitory neuron; Mic: microglia; Oli: oligodendrocyte; OPC: oligodendrocyte precursor cell.


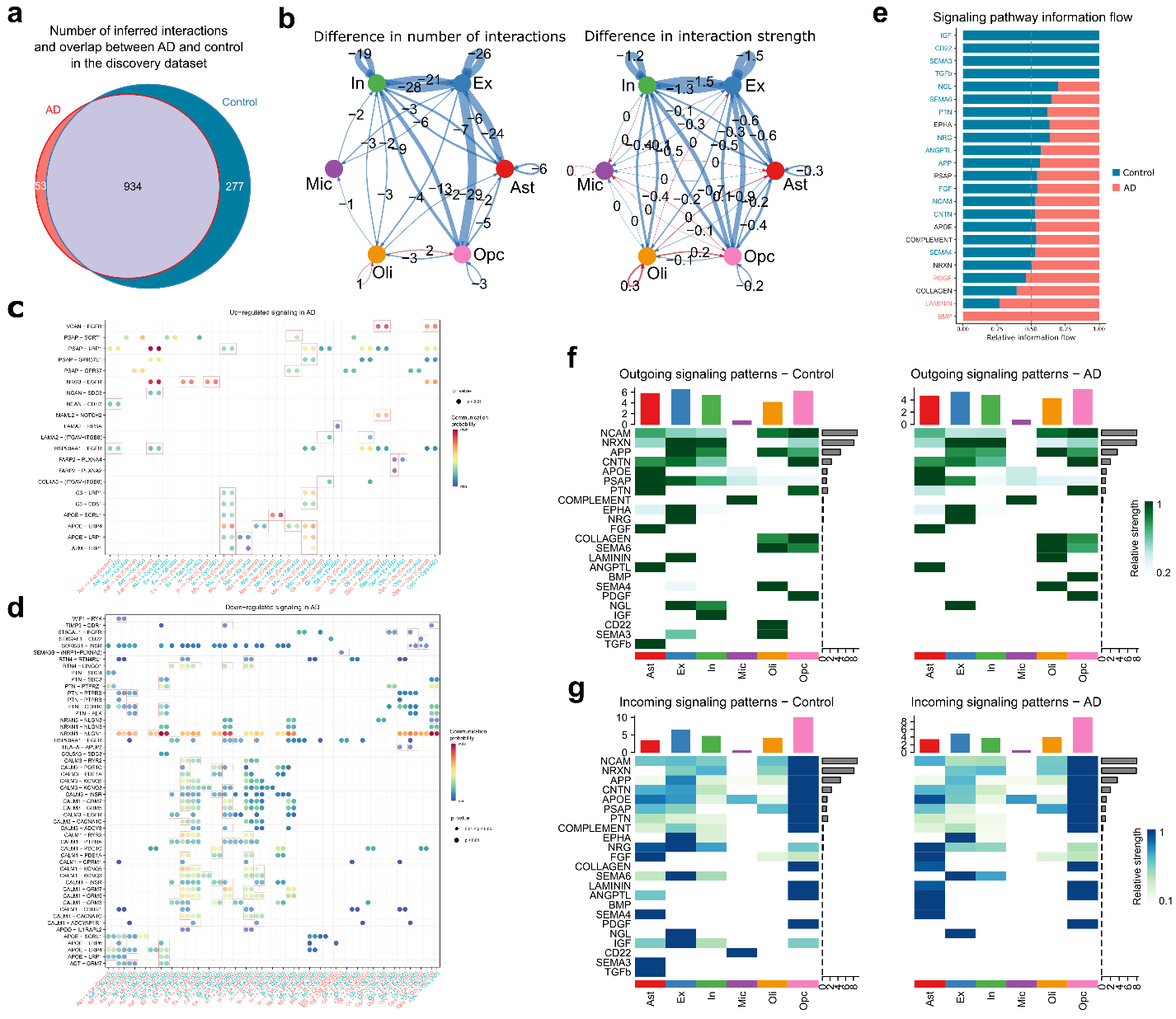


**Figure S2.** (**a**) The Venn diagram shows the number of inferred ligand-receptor (LR) interactions and overlaps between AD and control in the discovery dataset. (**b**) The difference in the number of intercellular interactions and difference in interaction strength between AD and controls across major cell types in the brain. (**c**) Bubble plot shows the cell-type-specific upregulated LR pairs in AD. LR interactions with ligand genes differentially expressed between AD and control were highlighted and defined as dysregulated LR interactions. (**d**) Bubble plot shows the cell-type-specific downregulated LR pairs in AD. LR interactions with ligand genes differentially expressed between AD and control were highlighted and defined as dysregulated LR interactions. (**e**) The relative information flow within intercellular signaling pathways, defined as the summation of interaction strength within the pathway, quantitatively compared between AD and controls. (**f**) The cell type-specific outgoing signaling patterns in controls (left) and AD (right). The color represents the relative outgoing signaling strength of each signaling pathway. (**g**) Cell type-specific incoming signaling patterns in controls (left) and AD (right). The color represents the relative incoming signaling strength of each signaling pathway.


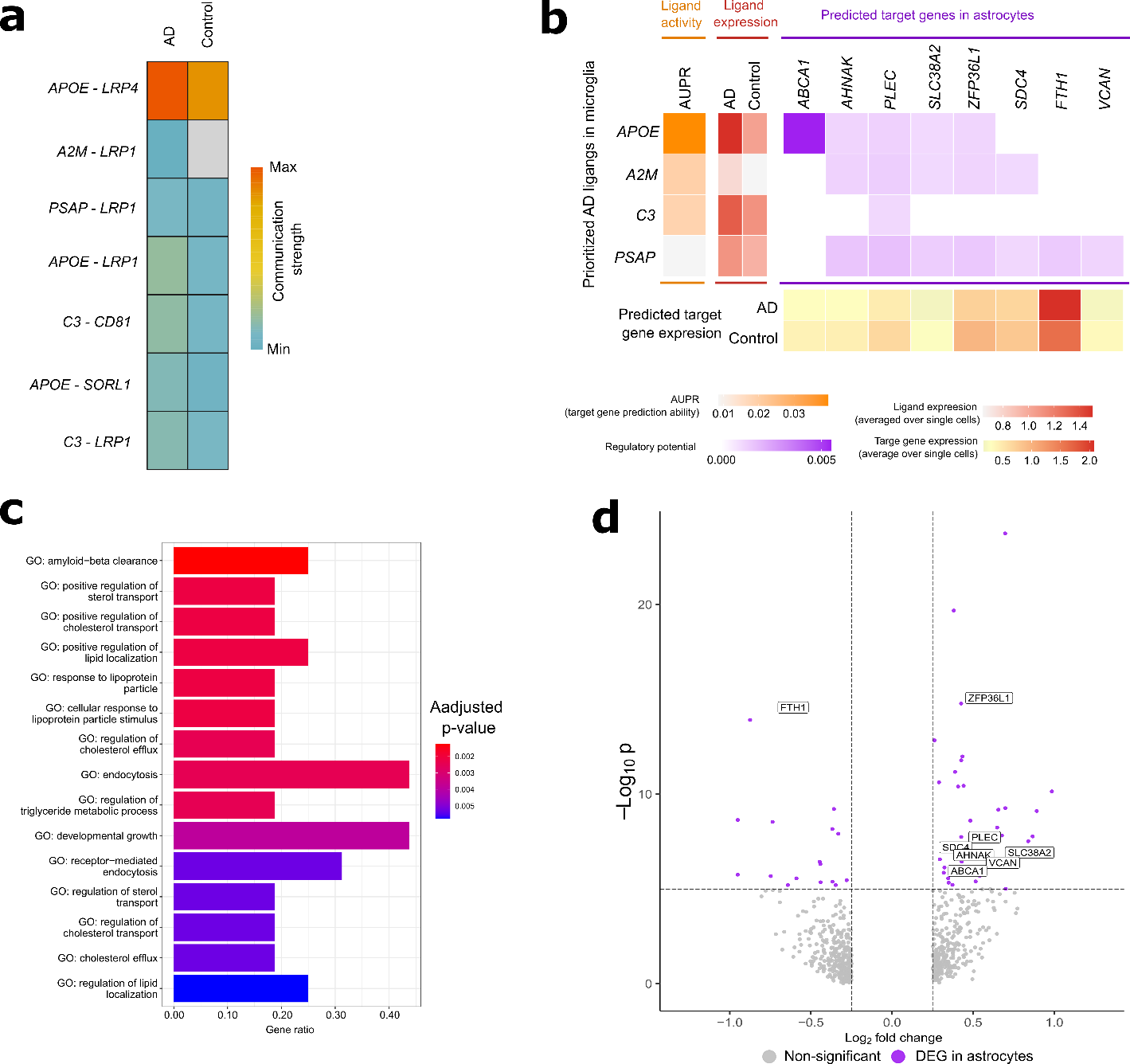


**Figure S3.** Ligand–target gene analysis of dysregulated ligand-receptor (LR) pairs from microglia to astrocytes. (**a**) Communication strength of dysregulated LR pairs from microglia to astrocyte in AD and controls. (**b**) The heatmap depicts the regulatory potential scores (purple) of each ligand gene of dysregulated LR pairs in microglia to differentially expressed genes (DEGs) in astrocytes. The ligand genes were ranked by the area under the precision-recall curve (AUPR, orange) and level of expression in astrocytes (red). The expression level of the predicted target gene in excitatory neurons is shown (yellow to red). (**c**) Bar plot shows the top 15 Gene Ontology Biological Processes significantly enriched in dysregulated LR pairs between microglia and astrocytes and their predicted target genes. (**d**) Volcano plot depicts the DEGs in astrocytes in the discovery dataset.
